# Etherization

**Published:** 1847-10

**Authors:** 


### ETHERIZATION.

Our Foreign Correspondent has favored us with a translation of the elaborate memoir of Baron Dubois on the subject of etherization in obstetrics. It will be found exceedingly interesting. In another department, we republish the report of a case in which the *protoxide of nitrogen* was successfully used to destroy sensibility before a painful surgical operation. We wish to call the attention of our readers particularly to this case, in the hope that similar trials may be made with the nitrous oxide, which possesses decided advantages over the vapor of ether in being free from all narcotic properties. If found to be equally effectual in annulling the pain of operations, it will probably supersede the ether.

